# Outcomes of concomitant antiobesity medication use with endoscopic sleeve gastroplasty in clinical US settings

**DOI:** 10.1016/j.obpill.2024.100112

**Published:** 2024-05-09

**Authors:** Khushboo Gala, Wissam Ghusn, Vitor Brunaldi, Christopher McGowan, Reem Z. Sharaiha, Daniel Maselli, Brandon Vanderwel, Prashant Kedia, Michael Ujiki, Eric Wilson, Eric J. Vargas, Andrew C. Storm, Barham K. Abu Dayyeh

**Affiliations:** a Division of Gastroenterology and Hepatology, Mayo Clinic, Rochester, MN, USA; b True You Weight Loss, Cary, NC, USA; c Division of Gastroenterology and Hepatology, Weill Cornell Medicine, New York, USA; d Eviva, Shoreline, WA, USA; e Methodist Dallas Medical Center, Dallas, TX, USA; f NorthShore University Health System, Evanston, IL, USA; g University of Texas Health Science Center – Houston, Houston, TX, USA

**Keywords:** Endoscopic sleeve gastroplasty, Antiobesity medications, Obesity, Bariatric endoscopy, GLP-1 receptor agonists, Weight loss

## Abstract

**Background:**

To evaluate the weight loss outcomes of the large US cohort of patients undergoing endoscopic sleeve gastroplasty (ESG) with or without concomitant anti-obesity (AOM) use.

**Methods:**

We performed a retrospective analysis of adult patients who underwent ESG from seven different sites, from January 1, 2020 to November 30, 2022. Percent total body weight loss (%TBWL) and %excess weight loss (%EWL) were calculated based on baseline weight at the procedure. Medication use was considered if the subject received a prescribed AOM during the study period. SPSS (version 29.0) was used for statistical analyses.

**Results:**

A total of 1506 patients were included (1359 (90.2 %) no AOM use and 147 (9.8 %) AOM use). Patients who were on an active AOM at the time of the procedure had a significantly lower TBWL% as compared to patients not on AOMs at 6 months. At the 24-month visit, patients who were prescribed AOMs after the 12-month visit had a significantly higher TBWL% and EWL% as compared to patients who were on active AOM at the time of the procedure. There was no significant difference between classes of medications at any time point, however, patients on a GLP-1RA had a trend towards improved weight loss at 18 and 24 months.

**Conclusion:**

In this large, real-world cohort of patients from the United States, data signal that with the use of pharmacotherapy at the appropriate time, patients can achieve optimal results.

## Graphical abstract

**Figure:**
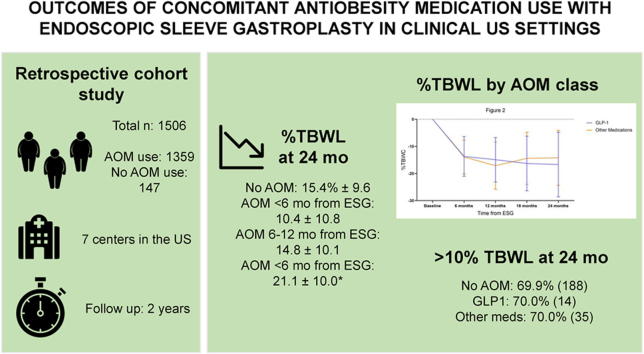

## Abbreviations

AOM: anti-obesity medications
EBT: endoscopic bariatric therapies
GLP-1RA: glucagon-like-1 receptor agonists
ESG: endoscopic sleeve gastroplasty
%TBWL: percentage total body weight loss
%EWL: percentage excess weight loss

## 1 Introduction

There has been an exponential rise in the incidence of obesity globally [1], however, advancements in therapeutic options have been slower than the pace of the disease. Limited options are available for the management of obesity, including anti-obesity medications (AOM), bariatric surgery and endoscopic bariatric therapies (EBT) [2]. As seen with other chronic diseases like hypertension and diabetes, using a combination of several available therapeutic options is often necessary to obtain optimal goals. For instance, AOMs have been found to be effective adjuncts for post-bariatric weight recurrence [3]. Several different medications have been used, with data suggesting that glucagon-like-1 receptor agonists (GLP-1RA) may be most effective in these cases [4].

Over the last decade, EBTs have emerged as an effective treatment of obesity, with a favorable safety profile [5]. Endoscopic sleeve gastroplasty (ESG) is a novel technique that involves the plication of the gastric body using an endoscopic suturing device [6]. ESG yields a mean percentage total body weight loss (%TBWL) >15 %, however, heterogeneity in response to treatment exists [7]. In clinical practice, providers often use AOMs in addition to ESG to reach weight loss targets. We examine the real-world use of AOMs in conjunction with ESG and evaluate the weight loss outcomes in the largest American cohort described to date.

## 2 Materials and methods

### 2.1 Study design and eligibility criteria

We performed a retrospective analysis of patients who underwent ESG at 7 different sites across the USA from January 2013 through August 2022. These sites included academic and private institutions, and procedures were performed by gastroenterologists and bariatric surgeons. The primary site institutional review board approved the study and waived the need for informed consent owing to its minimal-risk nature. IRB approval was also obtained at other sites. All adult patients who had undergone ESG using a standard technique with full thickness suturing using the Overstitch™ device (Apollo Endosurgery, Austin, Texas, USA) with the primary goal of weight loss were included.

### 2.2 Data collection and study end points

Patient demographic and medical information were abstracted from the electronic medical records. Indications and technical details of each procedure were collected. Baseline weight was defined as weight on the day of the intervention. Weight was recorded at baseline, 6, 12, 18, and 24 months after the procedure. Both intraprocedural and post-procedural adverse events (AEs) were recorded. %TBWL and percentage excess weight loss (%EWL, based upon BMI=25 kg/m^2^) were calculated based on baseline weight. Responders to treatment were defined as reaching a predetermined threshold for %TBWL at 12 and 24 months. Each responder group included the number of patients that satisfy those criteria. Completers are defined as those patients who had data collected at the 24-month visit.

AOM use was considered if the patient had an active prescribed weight loss medication at the time of the procedure or received a prescribed weight loss medication co-therapy during the first 2 years of treatment (length of study follow-up). If AOM therapy was started after the procedure, then one of 3 groups was reported for the patient: less than 6 months from the procedure, between 6 and 12 months from the procedure, and after 12 months. These groups are described as such: X < 0 days (ongoing at time of procedure), X < 6 months (started less than 6 months after procedure), 6 months ≤ X < 12 months (started between 6 and 12 months from the procedure), and X ≥ 12 months (started after 12 months). AOMs were placed into two different groups: GLP-1RA or others (phentermine, phentermine-topiramate, bupropion-naltrexone, orlistat). If a patient was prescribed an active medication(s) in each group near the same time, the patient was classified as taking GLP-1RA only since this medication will have the most pronounced impact on weight loss.

The primary aim of our study was to assess and compare weight loss outcomes in patients with and without AOM use. The secondary outcomes included comparing weight outcomes based on time from procedure to start AOM therapy, comparing weight outcomes based on medication classes, and analyzing treatment responders (%TBWL ≥ 10) at 24 months.

### 2.3 Statistical analysis

All continuous data are summarized as the means and 95 % confidence intervals (CI). For each follow-up visit, differences between groups with respect to time to starting AOM therapy were evaluated with one-way ANOVA. If a statistical difference was identified, the comparisons that were significantly different were obtained based upon Bonferroni Correction during analysis. Significance was defined as p < 0.05. Mean difference of response was defined as mean GLP-1RA – mean other medications. Imputation methods were used to evaluate the impact of missing data from the results. Last observation carried forward (LOCF) reported the responder level at the last recorded visit, that is if the patient did not return for any visit, the patient was not considered a responder. Best case scenario was defined such that those patients that had missing data were considered a responder at 10 % TBWL while worst case scenario was defined as patients that had missing data were not considered responders. A subanalysis of weight outcomes of a 1:1 matched cohort (based on age, sex, and baseline BMI) was also reported. SPSS (Version 29.0) was used for statistical analyses.

## 3 Results

### 3.1 Baseline characteristics

Our cohort comprised 1506 patients, who were distributed as follows: 1302 subjects with no AOM use (86.5 %) and 204 (13.5 %) subjects with AOM use. The cohort was predominantly female (84.5 %) and Caucasian (69.6 %), with a mean age of 45.68 ± 10.25 years. Mean weight and BMI at baseline were 107.3 ± 21.42 kg and 38.43 ± 6.22 kg/m^2^ respectively.

Baseline demographics with respect to AOM use are reported in Table 1. There was a higher proportion of African Americans and females in the group that did not receive AOMs. Patients who received AOMs were significantly younger than patients who did not receive AOMs (mean age 43.57 ± 10.2 years compared to 45.90 ± 10.23 years, two-sample *t*-test p=0.009). There was no difference between the two groups with respect to obesity class (Chi-square p-value = 0.772). There was a significant difference in the percentage of patients who received AOMs by provider type (Chi-square p-value <0.001). Surgeons prescribed AOM more frequently than gastroenterologists (15.7 % compared with 8.7 % respectively). There were no differences in baseline anthropomorphic data, including weight, height, and BMI.

**Table 1:** Baseline characteristics, by AOM use.

Description	No AOM (N=1359)	AOM (N=147)	Total (N=1506)
Sex	∗		∗
Male	205 (15.1 %)	28 (19.0 %)	233 (15.5 %)
Female	1153 (84.9 %)	119 (81.0 %)	1272 (84.5 %)
Race			
N	1206	110	1316
Caucasian	837 (69.4 %)	79 (71.8 %)	916 (69.6 %)
African American	208 (17.2 %)	6 (5.5 %)	214 (16.3 %)
Asian	20 (1.7 %)	3 (2.7 %)	23 (1.7 %)
Hispanic	24 (2.0 %)	1 (0.9 %)	25 (1.9 %)
Other	25 (2.1 %)	2 (1.8 %)	27 (2.1 %)
Not Reported	92 (7.6 %)	19 (1.4 %)	111 (8.4 %)
Obesity Class			
Class I	450 (33.1 %)	51 (34.7 %)	501 (33.3 %)
Class II	491 (36.1 %)	55 (37.4 %)	546 (36.3 %)
Class III	418 (30.8 %)	41 (27.9 %)	459 (30.5 %)
Provider Type			
Surgeon	198 (14.6 %)	37 (25.2 %)	235 (15.6 %)
Gastroenterologists	1161 (85.4 %)	110 (74.8 %)	1271 (84.4 %)
Age (years)		∗	∗
Mean (SD)	45.90 (10.23)	43.57 (10.25)	45.68 (10.25)
Height (m)			
Mean (SD)	1.67 (0.09)	1.68 (0.09)	1.67 (0.09)
Weight (kg)			
Mean (SD)	107.30 (21.57)	107.40 (20.01)	107.30 (21.42)
Ideal Weight (kg)			
Mean (SD)	69.70 (7.34)	70.50 (7.62)	69.70 (7.37)
Excess Weight (kg)			
Mean (SD)	37.60 (18.08)	36.80 (16.14)	37.60 (17.90)
BMI (kg/m^2^)			
Mean (SD)	38.47 (6.30)	38.02 (5.42)	38.43 (6.22)

### 3.2 Weight loss outcomes by time of AOM therapy

**Fig. 1:**
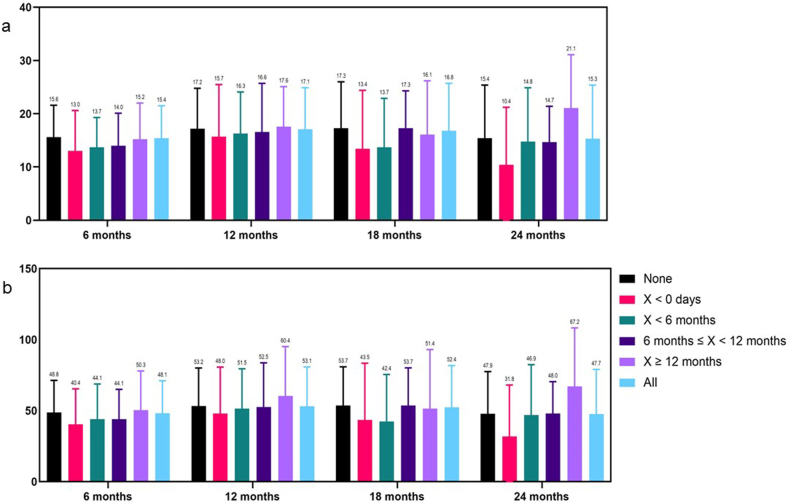
a and b: %Total Body Weight Loss (TBWL) and %Excess Weight Loss (EWL) outcomes by group and time from procedure.

Weight loss parameters by time from procedure to start AOM therapy are reported for BMI (Supplementary Table 3S), %TBWL, and %EWL (Fig. 1a and b; detailed analyses in Supplementary Tables 1S and 2S). No differences were identified for BMI at any follow-up visit based on if or when AOMs were initiated. With respect to TBWL% and EWL%, only those patients who received AOMs after 6-month follow-up did not demonstrate any weight gain at any follow-up visit. A statistical difference was identified for TBWL% at 6-month (p=0.003) and 24-month (p=0.014) follow-up visits, and EWL% at 24-month (p=0.007) follow-up visits. At the 6-month visit, patients who were on an active AOM at the time of the procedure had a significantly lower TBWL% as compared to patients who did not undergo medication co-therapy during the study. At the 24-month visit, patients that were prescribed AOM after the 12-month visit had a significantly higher TBWL% and EWL% as compared to patients that were on active AOM at the time of the procedure. No other comparisons were statistically significant.

### 3.3 Weight loss outcomes by AOM class

**Fig. 2:**
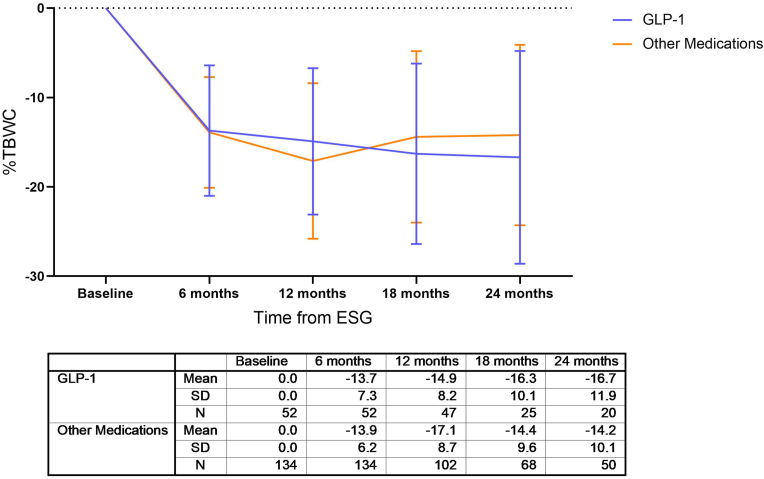
%Total Body Weight Change (TBWC) outcomes by type of AOM.

Weight loss parameters by time from procedure for patients that received AOM therapy are reported by medication class for %TBWL (Fig. 2, illustrated as Total Body Weight Change (TBWC); detailed analyses including BMI, %TBWL and %EWL in Supplementary Tables 4S, 5S, 6S). There was no significant difference between class of medications at any time point, however, patients on a GLP-1RA had a trend towards improved weight loss at 18 and 24 months, compared to other medications.

### 3.4 Treatment response at end of study period

At 24 months, treatment responders (%TBWL≥10) by AOM use (Table 2) and time to AOM from procedure (Table 3) were evaluated. We have described the response rate for completers, as well as imputation analyses including LOCF, best- and worst-case scenarios. There was no difference identified between groups based on AOM use (p-value = 1.000), implying that the percentage of responders to treatment with the use of medication is identical to that achieved without medication. This held true when adjusted for baseline sex, age, and BMI (p-value 0.05). Similarly, the percentage of responders to treatment was similar no matter if or when weight loss medication was prescribed (p-value = 0.088 for completers).

**Table 2:** Responders by Anti-Obesity Medication Class at 24 months.

Analysis Population		AOM Class		
		No AOM	GLP-1	Non-GLP-1 AOMs
Completers	N	269	20	50
	% (n)	69.9 % (188)	70.0 % (14)	70.0 % (35)
Last Observation Carried Forward	N	1302	58	146
	% (n)	61.8 % (804)	67.2 % (39)	69.9 % (102)
Best Case Scenario	N	1302	58	146
	% (n)	93.8 % (1221)	89.7 % (52)	89.7 % (131)
Worst Case Scenario	N	1302	58	146
	% (n)	14.4 % (188)	24.1 % (14)	24.0 % (35)

**Table 3:** Responders by Medication Start Time at 24 months.

Analysis Population		Medication Start Time				
		None	X < 0 days	X < 6 months	6 months ≤ X < 12 months	X ≥ 12 months
Completers	N	269	25	15	11	19
	% (n)	69.9 % (188)	52.0 % (13)	66.7 % (10)	81.8 % (9)	89.5 % (17)
Last Observation Carried Forward	N	1302	60	58	49	37
	% (n)	61.8 % (804)	53.3 % (32)	74.1 % (43)	79.5 % (39)	73.0 % (27)
Best Case Scenario	N	1302	60	58	49	10
	% (n)	93.8 % (1221)	80.0 % (48)	91.4 % (53)	95.9 % (47)	94.6 % (35)
Worst Case Scenario	N	1302	60	58	49	10
	% (n)	14.4 % (188)	21.7 % (13)	17.2 % (10)	18.4 % (9)	45.9 % (17)

### 3.5 Weight outcomes of matched cohort

A 1:1 matched cohort based on age and sex was reviewed (age in no AOM vs AOM groups: 44.01 ± 9.8 vs 43.57 ± 10.25; sex, %female in no AOM vs AOM groups: 84.6 vs. 84.9). Weight loss at 6 months was significantly different (15.7 ± 5.9 vs. 14.0 ± 6.6, p < 0.01), however, similar %TBWL was noted at 12, 18, and 24 months (12 months: 17.6 ± 8.4 vs. 16.8 ± 9.0, p = 0.37; 18 months: 15.8 ± 9.0 vs. 15.2 ± 9.6, p = 0.57; 24 months: 15.1 ± 9.2 vs. 14.7 ± 10.6, p = 0.73).

## 4 Discussion

We present one of few studies focused on outcomes of ESG and AOMs, and the first to our knowledge published from US data. In this large cohort of patients from across the United States, there are no significant differences in weight loss outcomes in patients who have undergone ESG and received or not received concomitant pharmacotherapy. Being a real-world study, AOMs were prescribed for patients who reported weight regain or poor weight loss, and generally not with an intention to augment weight outcomes. In this context, it was interesting to note that this held true and patients who started AOMs at different time points from the procedure had non-inferior weight outcomes to those not on AOMs. These data signal that using AOMs may help non-responders achieve similar outcomes to responders.

Notably, it was seen that the peak %TBWL seen in patients who were started on AOMs after ESG occurred about 6–12 months after starting the medication. This was most clearly evidenced by patients in whom AOMs were started at or after 12 months, who achieved maximal weight loss, crossing 20 % TBWL, at 24 months. This was significantly higher than weight loss for other groups at this point; and indeed, the highest %TBWL was seen within all subgroups (described in Fig. 1 and Supplementary Tables 1S and 2S). This was also noted in the completer's analysis at 24 months, which demonstrates that almost 90 % of patients who started AOMs ≥ 12 months achieved a %TBWL of ≥10 %; compared to 70 % of patients not on AOMs, and only 50 % of patients who were on AOMs prior to ESG. This may be due to the fact that the maximal effect of the ESG is seen at 6–12 months, after which there may be a plateauing of weight; the addition of an AOM at that time likely potentiates and sustains weight loss. It was also noteworthy that patients who were on an AOM prior to ESG had the lowest %TBWL of all groups, and the lowest response rate at 24 months. A possible explanation for this may be that these patients have already achieved some degree of weight loss, hence limiting the amount of weight loss that can be induced by the ESG (although this is not reflected by their BMI at different time points); or that they had maximized the efficacy of AOM prior to ESG. It is possible that these may be patients who have a different phenotype of obesity that may be more resistant to weight loss, and hence have been on an AOM even before undergoing ESG.ESG and GLP-1RAs share a common mechanism of weight loss (delayed gastric emptying), and hence for patients on a GLP-1 agonist prior to ESG, they may have already reached a plateau in terms of gastric delay and hence may have lesser weight loss. This heterogeneity of weight loss and different trajectories of weight loss have been described in bariatric surgery literature previously [[8], [9], [10]], and needs further study in the realm of precision medicine.

We also reviewed weight loss based on type of medication used. GLP-1RAs have emerged as the class of AOMs yielding the highest mean %TBWL. Real-world and trial data describe a mean %TBWL of about 10 % with the use of semaglutide, currently the most popular GLP-1RA in the United States [11,12]. Although we did note a trend towards higher weight loss with GLP1-RA used in conjunction with ESG compared to other medications at 18 and 12 months, this was not statistically significant. Given our sample size, we could not perform meaningful head-to-head comparisons between specific other types of medications and GLP-1RAs.

There is very limited data on the use of AOMs in combination with ESG. Badurdeen et al. (Brazil) described a retrospective review of 26 matched pairs of patients who underwent either ESG alone, or ESG with liraglutide started at 5 months, and found a significantly higher %TBWL and reduction in percent body fat at 12 months in the latter [13]. Hoff et al. (Brazil) showed similar results with addition of semaglutide at 5 months in 27 cases and 28 controls, in the form of a double blinded, sham controlled prospective study [14]. We postulate a concept of “proactive” versus “reactive” AOM use – our study focuses on reactive AOM use (prescribed in cases with inadequate weight loss or weight recurrence), as compared to these studies that describe proactive AOM (prescribed in all cases). Proactive AOM likely yields higher weight loss, as reflected in results of the abovementioned studies compared to our study. Further prospective data is required to uncover this concept of proactive and reactive AOM use.

Our study has several strengths. It has the largest sample size and longest follow-up of the limited data present on AOMs and ESG. The use of this cohort of American patients who have undergone ESG, derived from a variety of practice and provider types, lends a high level of generalizability to the data for readers in USA. A recent paper from our group describes minor variations in technique between gastroenterologists and surgeons, with overall similar results after the procedure and safety [15]. It is also interesting to note higher AOM use among surgeons compared with gastroenterologists; although the exact reason for this is unclear, we hypothesize that practice setting (larger bariatric practices) and higher experience and comfort with use of AOMs (may also be using in post-bariatric surgery weight regain cases) plays into this. We have a follow up period of 2 years and have used multiple statistical models to compensate for the attrition of patients. The study is limited by its retrospective nature, as well as data gathered by several sites. Granular details of indications of AOM use and adverse events related to AOMs were not captured consistently in our database, and hence not reported. There is a possibility of recall bias with exact dates of medication initiation and termination, and lastly, the fact that any information that was not reported by the patient or entered into the EMR portal by the health care professional could potentially be missed.

## 5 Conclusions

Obesity care is going through an exciting and revolutionary period, with several new drugs and procedures in the pipeline. Our data signal that with use of pharmacotherapy at the appropriate time, patients can achieve target results. Using different therapeutic modalities in combination seems to be the key to optimizing and augmenting outcomes and minimizing adverse events to achieve significant and sustainable weight loss.

1. Patients who were prescribed AOMs after the 12-month visit had a significantly higher TBWL% and EWL% at 24 months, as compared to patients who were on active AOM at the time of the procedure.
2. Patients on a GLP-1RA had a trend towards improved weight loss at 18 and 24 months.

## Author contribution

The concept of the submission was by KG and BA. Statistical analysis and data curation was performed by KG, WG and VB. KG wrote the first draft. All authors reviewed, edited, and approved the final submission and publication.

## Funding

Beyond payment to the research staff by respective universities, this research did not receive any specific grant from funding agencies in the public, commercial, or not-for-profit sectors.

## Ethical adherence and ethical review

The primary site institutional review board approved the study and waived the need for informed consent owing to its minimal-risk nature. IRB approval was also obtained at other sites.

## Disclosures

Andrew C. Storm has research grants from Apollo Endosurgery, Boston Scientific, Endogenex, Enterasense, OnePass, and is a consultant for Apollo Endosurgery, Boston Scientific, Endogenex, Endo-TAGSS, MGI Medical, Olympus, Intuitive, Medtronic, Microtech.

Michael Ujiki is a board member for Boston Scientific, is a paid consultant for Olympus and Cook, and receives payment for lectures from Medtronic, Gore and Erbe.

Prashant Kedia is a consultant for Boston Scientific, Medtronic, and Olympus.

Reem Z. Sharaiha is a consultant for Boston Scientific, Cook Medical, and Lumendi.

Brandon VanderWel is a consultant for Apollo Endosurgery.

Daniel Maselli is a consultant for Apollo Endosurgery.

Barham K. Abu Dayyeh is a consultant for DyaMx, Boston Scientific, USGI Medical, and Endo-TAGSS; gets research support from Boston Scientific, USGI Medical, Apollo Endosurgery, Spatz Medical, GI Dynamics, Cairn Diagnostics, Aspire Bariatrics, and Medtronic; is a speaker for Johnson and Johnson, Endogastric Solutions, and Olympus.

Other authors do not have a conflict of interest or disclosures.

## Declaration of Artificial Intelligence (AI) and AI-assisted technologies

No AI or AI-assisted technologies were used for this submission.
