# Corrigendum to “Outcomes of concomitant antiobesity medication use with endoscopic sleeve gastroplasty in clinical US settings” [Obes. Pillars, Volume 11, September 2024, 100112]

**DOI:** 10.1016/j.obpill.2024.100116

**Published:** 2024-06-29

**Authors:** Khushboo Gala, Wissam Ghusn, Vitor Brunaldi, Christopher McGowan, Reem Z. Sharaiha, Daniel Maselli, Brandon Vanderwel, Prashant Kedia, Michael Ujiki, Eric Wilson, Eric J. Vargas, Andrew C. Storm, Barham K. Abu Dayyeh

**Affiliations:** a Division of Gastroenterology and Hepatology, Mayo Clinic, Rochester, MN, USA; b True You Weight Loss, Cary, NC, USA; c Division of Gastroenterology and Hepatology, Weill Cornell Medicine, New York, USA; d Eviva, Shoreline, WA, USA; e Methodist Dallas Medical Center, Dallas, TX, USA; f NorthShore University Health System, Evanston, IL, USA; g University of Texas Health Science Center – Houston, Houston, TX, USA

The authors regret two typographical errors in the graphical abstract:

1) in the left column – “AOM use: 147, no AOM use: 1359”;
2) middle column, second-to-last line, should be “AOM >6 mo from ESG”.

The authors would like to apologise for any inconvenience caused.

