# Supplementary material for: Outcomes of concomitant antiobesity medication use with endoscopic sleeve gastroplasty in clinical US settings
Source: Obes Pillars. 2024 May 9;11:100112. doi: 10.1016/j.obpill.2024.100112 (PMC11145356; doi:10.1016/j.obpill.2024.100112)
Supplement: Multimedia component 1 [file mmc1.docx]

**Supplementary material**

Table 1S: %TBWL Outcomes by Group and Time from Procedure

| **Time from Procedure** | **Medication Start Time with Respect to Procedure** | | | | | |
| --- | --- | --- | --- | --- | --- | --- |
|  | **None** | **X < 0 days** | **X < 6 months** | **6 months ≤ X < 12 months** | **X ≥ 12 months** | **All** |
| 6 months |  |  |  |  |  |  |
| Mean (STD) | 15.6 (5.9856) | 12.95 (7.5536) | 13.7 (5.6290) | 14.0 (6.1475) | 15.2 (6.7541) | 15.4 (6.1075) |
| N | 1002 | 52 | 53 | 46 | 35 | 1188 |
| Min, Max | -5.3, 44.3 | -4.9, 40.0 | -2.3, 24.8 | 1.4, 29.7 | 0.0, 29.3 | -5.3, 44.3 |
| 95% CI | 15.3, 16.0 | 10.8, 15.1 | 12.2, 15.3 | 12.2, 15.9 | 12.9, 17.5 | 15.0, 15.7 |
| 12 months |  |  |  |  |  |  |
| Mean (STD) | 17.2 (7.6255) | 15.7 (9.7802) | 16.3 (7.8397) | 16.6 (9.0575) | 17.6 (7.5443) | 17.1 (7.7862) |
| N | 802 | 41 | 43 | 36 | 29 | 951 |
| Min, Max | -9.6, 49.7 | 0.0, 38.9 | -1.0, 37.9 | 0.0, 34.3 | 4.3, 35.3 | -9.6, 49.7 |
| 95% CI | 16.7, 17.7 | 12.6, 18.7 | 13.9, 18.7 | 13.5, 19.6 | 14.7, 20.5 | 16.6, 17.6 |
| 18 months |  |  |  |  |  |  |
| Mean (STD) | 17.3 (8.6560) | 13.4 (10.9795) | 13.7 (9.2183) | 17.3 (7.0494) | 16.1 (10.0520) | 16.8 (8.9038) |
| N | 390 | 28 | 22 | 16 | 27 | 483 |
| Min, Max | -7.5, 48.0 | -10.5, 34.6 | -4.6, 31.6 | 1.8. 30.0 | 0.9, 37.2 | -10.5, 48.0 |
| 95% CI | 16.4, 18.2 | 9.1, 17.6 | 9.6, 17.8 | 13.5, 21.1 | 12.1, 20.0 | 16.0, 17.6 |
| 24 months |  |  |  |  |  |  |
| Mean (STD) | 15.40 (9.9500) | 10.4 (10.7719) | 14.8 (10.1424) | 14.7 (6.6954) | 21.1 (10.0361) | 15.3 (10.0701) |
| N | 269 | 25 | 15 | 11 | 19 | 339 |
| Min, Max | -11.3, 50.0 | -12.4, 34.1 | -1.5, 34.3 | 6.0, 26.0 | 2.0, 41.1 | -12.4, 50.0 |
| 95% CI | 14.2, 16.6 | 5.9, 14.9 | 9.2, 20.4 | 10.2, 19.2 | 16.3, 25.9 | 14.3, 16.5 |

Table 2S: %EWL Outcomes by Group and Time from Procedure

| **Time from Procedure** | **Medication Start Time with Respect to Procedure** | | | | | |
| --- | --- | --- | --- | --- | --- | --- |
|  | **None** | **X < 0 days** | **X < 6 months** | **6 months ≤ X < 12 months** | **X ≥ 12 months** | **All** |
| 6 months |  |  |  |  |  |  |
| Mean (STD) | 48.8 (22.5997) | 40.4 (25.0045) | 44.1 (24.7095) | 44.1 (20.8888) | 50.3 (27.7250) | 48.1 (22.9648) |
| N | 1002 | 52 | 53 | 46 | 35 | 1188 |
| Min, Max | -12.3, 151.7 | -20.5, 109.4 | -12.7, 119.3 | 4.0, 89.3 | 0.0, 143.2 | -20.5, 151.7 |
| 95% CI | 47.4, 50.2 | 33.4, 47.3 | 37.3, 50.9 | 37.9, 50.3 | 40.8, 59.9 | 46.8, 49.4 |
| 12 months |  |  |  |  |  |  |
| Mean (STD) | 53.2 (26.9430) | 48.0 (32.6596) | 51.5 (28.0069) | 52.5 (31.2018) | 60.4 (34.7457) | 53.1 (27.6766) |
| N | 802 | 41 | 43 | 36 | 29 | 951 |
| Min, Max | -44.1, 228.3 | 0.0, 153.0 | -3.5, 111.4 | 0.0, 136.3 | 14.9, 193.6 | -44.1, 228.3 |
| 95% CI | 51.3, 55.1 | 37.7, 58.3 | 42.8, 60.1 | 41.9, 63.0 | 47.2, 73.6 | 51.3, 54.9 |
| 18 months |  |  |  |  |  |  |
| Mean (STD) | 53.7 (27.2307) | 43.5 (39.9060) | 42.4 (33.1161) | 53.7 (26.5064) | 51.4 (41.7346) | 52.4 (29.3554) |
| N | 390 | 28 | 22 | 16 | 27 | 483 |
| Min, Max | -34.8, 151.2 | -34.5, 148.2 | -15.7, 122.7 | 5.2, 107.5 | 3.6, 204.2 | -34.8, 204.2 |
| 95% CI | 50.9, 56.4 | 28.1, 59.0 | 27.7, 57.1 | 39.6, 67.8 | 34.9, 67.9 | 49.8, 55.1 |
| 24 months |  |  |  |  |  |  |
| Mean (STD) | 47.9 (29.5897) | 31.8 (36.2860) | 46.9 (35.5377) | 48.0 (22.4928) | 67.2 (41.0518) | 47.7 (31.3834) |
| N | 269 | 25 | 15 | 11 | 19 | 339 |
| Min, Max | -32.9, 193.9 | -41.0, 114.3 | -5.2, 132.9 | 17.0, 90.6 | 8.0, 196.3 | -41.0, 196.3 |
| 95% CI | 44.3, 51.4 | 16.8, 46.8 | 27.3, 66.6 | 32.8, 63.1 | 47.4, 87.0 | 44.4, 51.1 |

Table 3S: BMI Outcomes by Group and Time from Procedure

| **Time from Procedure** | **Medication Start Time with Respect to Procedure** | | | | | |
| --- | --- | --- | --- | --- | --- | --- |
|  | **None** | **X < 0 days** | **X < 6 months** | **6 months ≤ X < 12 months** | **X ≥ 12 months** | **All** |
| Baseline |  |  |  |  |  |  |
| Mean (STD) | 38.47 (6.32) | 38.79 (5.87) | 38.01 (4.49) | 38.02 (5.62) | 37.68 (6.34) | 38.43 (6.22) |
| N | 1302 | 60 | 58 | 49 | 37 | 1506 |
| 6 months |  |  |  |  |  |  |
| Mean (STD) | 32.68 (5.37) | 33.59 (5.76) | 32.73 (4.30) | 32.78 (4.80) | 32.15 (5.14) | 32.71 (5.31) |
| N | 1002 | 52 | 53 | 46 | 35 | 1188 |
| 12 months |  |  |  |  |  |  |
| Mean (STD) | 32.23 (6.05) | 32.39 (5.65) | 32.07 (4.64) | 31.78 (5.12) | 30.97 (5.30) | 32.18 (5.92) |
| N | 802 | 41 | 43 | 36 | 29 | 951 |
| 18 months |  |  |  |  |  |  |
| Mean (STD) | 31.63 (5.12) | 32.97 (6.28) | 33.07 (4.86) | 32.71 (6.59) | 32.46 (5.53) | 31.86 (5.25) |
| N | 390 | 28 | 22 | 16 | 27 | 483 |
| 24 months |  |  |  |  |  |  |
| Mean (STD) | 31.98 (5.02) | 34.37 (5.96) | 31.91 (4.59) | 31.82 (5.05) | 32.06 (5.14) | 32.06 (5.14) |
| N | 269 | 25 | 15 | 11 | 19 | 339 |

Table 4S: BMI for Subjects Receiving Medication by Type and Time from Procedure

| **Time from Procedure** | **GLP-1** | **Other Medications** | **Difference** |
| --- | --- | --- | --- |
| Baseline |  |  |  |
| Mean (STD) | 37.58 (5.4549) | 38.42 (5.5478) | -0.84 |
| N | 58 | 146 |  |
| Min, Max | 30.08, 54.87 | 30.00, 56.54 |  |
| 95% CI | 36.15, 39.01 | 37.51, 39.33 | -2.53, 0.85 |
| 6 months |  |  |  |
| Mean (STD) | 32.41 (5.1213) | 33.06 (4.9670) | -0.65 |
| N | 52 | 134 |  |
| Min, Max | 23.92, 47.35 | 22.59, 47.13 |  |
| 95% CI | 30.98, 33.83 | 32.21, 33.91 | -2.27, 0.97 |
| 12 months |  |  |  |
| Mean (STD) | 31.76 (5.0646) | 31.93 (5.2098) | 0.91 |
| N | 47 | 102 |  |
| Min, Max | 24.37, 46.20 | 19.79, 46.44 |  |
| 95% CI | 30.28, 33.25 | 30.90, 32.95 | -1.96, 1.64 |
| 18 months |  |  |  |
| Mean (STD) | 32.27 (5.4137) | 33.00 (5.8608) | -0.72 |
| N | 25 | 68 |  |
| Min, Max | 23.96, 46.49 | 19.20, 48.71 |  |
| 95% CI | 30.03, 34.51 | 31.58, 34.42 | -3.39, 1.94 |
| 24 months |  |  |  |
| Mean (STD) | 32.07 (6.2939) | 32.49 (5.3492) | -0.42 |
| N | 20 | 50 |  |
| Min, Max | 23.96, 47.35 | 19.64, 44.00 |  |
| 95% CI | 29.12, 35.02 | 30.97, 34.01 | -3.39, 2.56 |

Table 5S: %TBWL for Subjects Receiving Medication by Type and Time from Procedure

| **Time from Procedure** | **GLP-1** | **Other Medications** | **Difference** |
| --- | --- | --- | --- |
| 6 months |  |  |  |
| Mean (STD) | 13.7 (7.3214) | 13.9 (6.2407) | -0.2 |
| N | 52 | 134 |  |
| Min, Max | -4.9, 29.3 | -2.3, 40.0 |  |
| 95% CI | 11.7, 15.7 | 12.9, 15.0 | -2.3, 1.9 |
| 12 months |  |  |  |
| Mean (STD) | 14.9 (8.2054) | 17.1 (8.7256) | -2.2 |
| N | 47 | 102 |  |
| Min, Max | 0.0, 34.3 | -1.0, 38.9 |  |
| 95% CI | 12.5, 17.4 | 15.4, 18.8 | -5.2, 0.8 |
| 18 months |  |  |  |
| Mean (STD) | 16.3 (10.0949) | 14.4 (9.5648) | 1.9 |
| N | 25 | 68 |  |
| Min, Max | -0.6, 34.1 | -10.5, 37.2 |  |
| 95% CI | 12.1, 20.4 | 12.1, 16.7 | -2.6, 6.4 |
| 24 months |  |  |  |
| Mean (STD) | 16.7 (11.8876) | 14.2 (10.0546) | 2.5 |
| N | 20 | 50 |  |
| Min, Max | -4.9, 41.1 | -12.4, 35.7 |  |
| 95% CI | 11.2, 22.3 | 11.4, 17.1 | -3.1, 8.1 |

Table 6S. %EWL for Subjects Receiving Medication by Type and Time from Procedure

| **Time from Procedure** | **GLP-1** | **Other Medications** | **Difference** |
| --- | --- | --- | --- |
| 6 months |  |  |  |
| Mean (STD) | 45.0 (26.3462) | 44.0 (23.9116) | 1.0 |
| N | 52 | 134 |  |
| Min, Max | -20.5, 1119.3 | -12.7, 143.2 |  |
| 95% CI | 37.7, 52.3 | 39.9, 48.0 | -6.9, 9.0 |
| 12 months |  |  |  |
| Mean (STD) | 49.3 (27.7088) | 54.0 (33.0233) | -4.7 |
| N | 47 | 102 |  |
| Min, Max | 0.0, 111.4 | -3.5, 193.6 |  |
| 95% CI | 41.2, 57.4 | 47.5, 60.4 | -15.6, 6.3 |
| 18 months |  |  |  |
| Mean (STD) | 49.6 (30.3151) | 46.5 (38.9489) | 3.2 |
| N | 25 | 68 |  |
| Min, Max | -1.4, 120.4 | -34.5, 204.2 |  |
| 95% CI | 37.1, 62.1 | 37.0, 55.9 | -14.0, 20.3 |
| 24 months |  |  |  |
| Mean (STD) | 50.3 (34.6989) | 46.0 (39.1481) | 4.3 |
| N | 20 | 50 |  |
| Min, Max | -20.5, 111.4 | -41.0, 196.3 |  |
| 95% CI | 34.0, 66.5 | 34.9, 57.1 | -15.8, 24.3 |
